# Enhanced antioxidant phytochemicals and catalase activity of celery by-products by a combined strategy of selenium and PGPB under restricted N supply

**DOI:** 10.3389/fpls.2024.1388666

**Published:** 2024-09-13

**Authors:** Jacinta Collado-González, María Carmen Piñero, Ginés Otálora, Josefa López-Marín, Francisco M. del Amor

**Affiliations:** Department of Crop Production and Agri-Technology, Murcia Institute of Agri-Food Research and Development (IMIDA), Murcia, Spain

**Keywords:** antioxidant compounds, antioxidant enzyme activity, nitrogen dose, sustainable strategy, valorization of celery by-products

## Abstract

**Introduction:**

The reduction of N supplied combined with the use of biostimulants can be an efficient strategy that allows sustainable agriculture to achieve better economic, nutritional and environmental goals without reducing production. Moreover, the industrial processing of celery generates large amounts of waste. Therefore the purpose of this study was improve crop management strategies to reduce nitrate pollution while turning crop waste into value-added products for others sectors.

**Methods:**

Consequently, in this work twelve treatments were examined: three N nitrogen content in the nutrient solution (100% control, 60%, and 30%) combined with the inoculation of the roots with *Azotobacter salinestris*, and foliar application selenium solution (8 μM, Na_2_SeO_4_). The celery parts from plants grown under limited N dose showed a higher antioxidant activity and TPC (total phenolic compounds) content.

**Results and discussion:**

The antioxidant activity increased 28% in leaves and 41% in by-products and TPC improved 27% in leaves and 191% in by-products respect to the control. Besides, a significant reduction of β-carotene content (56%, 11% and 43% in petioles, leaves and by-products respect to the control, respectively) was obtained in plants fed with restricted dose of N. The catalase activity was not affected by N dose. The inoculation of the plants with *Azotobacter*, together with a reduced N dose, achieved a greater accumulation of all the parameters studied. This accumulation was maximum when Se was applied to the leaves compared with the control and depending on the celery part: TPC (121-450%); antioxidant activity (60-68%), of catalase activity (59% - 158%), and of pigments content (50-90%). These findings can boost the valorization of celery by-products as excellent source of bioactive compounds.

## 1 Introduction

In recent years, consumers have significantly changed their consumption habits, with an increase observed in their interest about the foods they eat, and the possible effects their diet may have on their health and the environment (Sánchez-Bravo et al., 2021). Celery (*Apium graveolens* L.) is a highly nutritious plant and part of a healthy diet, as it is considered a good source of biologically-active compounds, such carotenes, tocopherols, flavonoids, alkaloids, terpenoids, and phenolic acids (Al Aboody, 2021). Due to its broad biological activity, celery prevents and heals numerous ailments, including asthma, cardiovascular, liver, and spleen diseases, gastrointestinal disorders, increases male fertility, and different types of cancer (Golubkina et al., 2020; Beltrán Sanahuja et al., 2021). It has been reported that different parts of celery are edible and have broad biological activities (Al Aboody, 2021). However, during industrial exploitation, a large volume of agro-based waste (external petioles and leaves) is generated. These vegetable residues serve as a basis of feed for animals fertilizers (Beltrán Sanahuja et al., 2021). However, due to the great environmental benefits that may come from reusing such waste, there is a growing concern to find a process for their efficient and feasible recirculation, which may also contribute to the circular economy (Haque et al., 2023).

As reported by Munene et al (Munene et al., 2017), the nutritional and functional quality of plants can be enhanced by mineral treatment. Nitrogen (N) is a fundamental element for attaining adequate plant growth and development. With a suitable N application to the crop, not only can good production can be gained, but also a good accumulation of phytochemicals (Stefaniak et al., 2020). However, the excessive use of N fertilizer, an agronomic practice widely adopted by farmers, can result in significant decreases in these phytochemicals and an undesirable increase in the content of harmful compounds such as nitrates (Collado-González et al., 2023). This higher nitrate content in plants can have harmful effects on the ecosystem and human health (Collado-González et al., 2023). Therefore, in recent years, in order to achieve a good accumulation of bioactive compounds in plants, and at the same time, to alleviate the situation of contamination, the application of N fertilizers has been decreased (Munene et al., 2017). It is important to take into account that mineral nutrition affects both the primary and secondary metabolism of higher plants. In this sense, plants grown under nutrient-deficient conditions often show a high accumulation of secondary metabolites (Caretto et al., 2015). Previous studies have reported that deficiency of minerals such as phosphorus (P) and N promotes the accumulation or biosynthesis of antioxidant compounds (Munene et al., 2017; Stefaniak et al., 2020; Basit et al., 2022). On this point, it needs to be emphasized that a deficiency in N could also result in smaller plants, and a lower production and quality, caused by an N imbalance in plants (Collado-González et al., 2023; Munene et al., 2017). In this context, nitrogen biofertilization carried out with nitrogen-fixing bacteria can be considered as an interesting alternative to the use of traditional nitrogen fertilizers. Often, abiotic stresses, including salinity, drought and thermal stress, cause oxidative stress, registering an increase in the amounts of flavonoids and thermotolerant proteins (Liu et al., 2020; Yang et al., 2022). The existence of complex inter- and intra-relationships between antioxidants and plant tolerance to different types of abiotic stresses such as heavy metals and thermal stresses has been reported (Liu et al., 2020; Basit et al., 2022).

Bacteria of the *Azotobacter* genus are plant growth promoting bacteria (PGPB), and they are one of the groups of bacteria most used as biofertilizers (Jnawali et al., 2015). These PGPB stimulate plant growth, providing them with a greater tolerance against different types of abiotic stresses, such as nutritional stress (Collado-González et al., 2023). In previous works, it has been reported that inoculation of plants with this type of nitrogen-fixing bacteria (*Azotobacter*), under a reduced nitrogen regime, resulted in plants with improved quality and yields similar to those obtained through the use of nitrogen fertilizers (Aasfar et al., 2021). Thus, the application of *Azotobacter* bacteria, together with limited nitrogen fertilization, was advantageous for the environment.

Selenium (Se) is a micromineral that can be toxic for human in excessive doses (400 µg of Se per day); indeed, it is known as an “essential poison” (Genchi et al., 2023). However, Se is essential in important metabolic processes in humans, animals, and unicellular organisms (Schiavon et al., 2020). Specifically in humans, Se provides some health benefits, reducing the risk of suffering from certain ailments, including infections and immunosuppressed states, male infertility, thyroid disorders and cancer, Alzheimer’s disease and Parkinson’s disease, diabetes, Keshan disease, and endemic myxedema cretinism (De Oliveira Maia et al., 2020). For these reasons, there is currently a great global concern about diets deficient in Se and its potential impact on human health (Genchi et al., 2023). It is currently estimated that about one billion people in the world are affected by a diet low in Se (Genchi et al., 2023). Experts warn of a possible worsening of this harsh scenario as a consequence of climate change, as it could lead to a reduction in the Se content in the soil, mainly in agricultural areas (De Oliveira Maia et al., 2020). Regarding plants, previous studies have reported that selenium stimulates their growth and development and increases their tolerance to various types of abiotic and biotic stress (Ashraf Ganjouii et al., 2023). In addition, foods resulting from crops treated with selenium have a higher nutritional quality. For example, food crops that have been biofertilized with selenium are usually enriched with many phytochemicals beneficial to human health (Cunha et al., 2023).

The authors of this work start from the hypothesis that inoculation with PGPB and the application of selenium at adequate concentration should modify some parameters of the celery. It could improve its tolerance to stress caused by the application of a nutrient solution with a reduced supply of N and enhancing their health-promoting effects in all celery parts. Thus, it is also assumed that this strategy will also allow to take advantage of the celery by-products.

Considering the above, the objective of this work is to study how affect inoculation with the PGPB, *Azotobacter salinestris*, on the accumulation of antioxidant compounds, as well as enzymatic antioxidant activity, particularly catalase, in different celery parts (petioles, leaves, and by-products), under different N doses. The effect of the inoculation with this PGPB combined with the application of selenium in plants grown under different N doses on the mentioned parameters was also studied. Moreover, this study provides information on a new sustainable strategy shaped by the synergistic relationship between selenium at an adequate concentration, and inoculation with *Azotobacter salinestris*, in plants fed with a suitable dose of N. This strategy allows for the further valorization of celery by-products in order to promote the exploitation of these by-products in pharmaceutical and nutraceutical industries.

## 2 Materials and methods

### 2.1 Experimental design and treatments

#### 2.1.1 Experimental site and design

The experiments were laid out in two identical modules of a polycarbonate greenhouse located in Murcia, Spain (37°56’27.3”N, 1°08’01.8”W). Seventy-two celery plants cv. Gladiator (Babyplant S.L., Santomera, Murcia, Spain) were transplanted in plastic bags filled with only coconut fiber (Pelemix, Alhama de Murcia, Murcia, Spain) and randomly arrangement within each module. The separation between two consecutive plastic bags within a row was 33 cm, and the rows were 1 m apart. All the celery plants were grown under controlled conditions, with a day/night temperature regime of 28/15°C and relative humidity of 70%. A daily drainage control was carried out in order to ensure plant drainage greater than 35% to ensure that there is no precipitation of salts in the root environment. A modified Hoagland’s nutrient control solution, composed of Ca(NO_3_)_2_·4H_2_O (362.0 mg L^−1^), KNO_3_ (404.4 mg L^−1^), K_2_SO_4_ (131.1 mg L^−1^), MgSO_4_ 7H_2_O (123.2 mg L^−1^), H_3_PO_4_ (0.101 mL) and micronutrients, was used for the irrigation.

#### 2.1.2 Treatments and procedure

**Figure 1:**
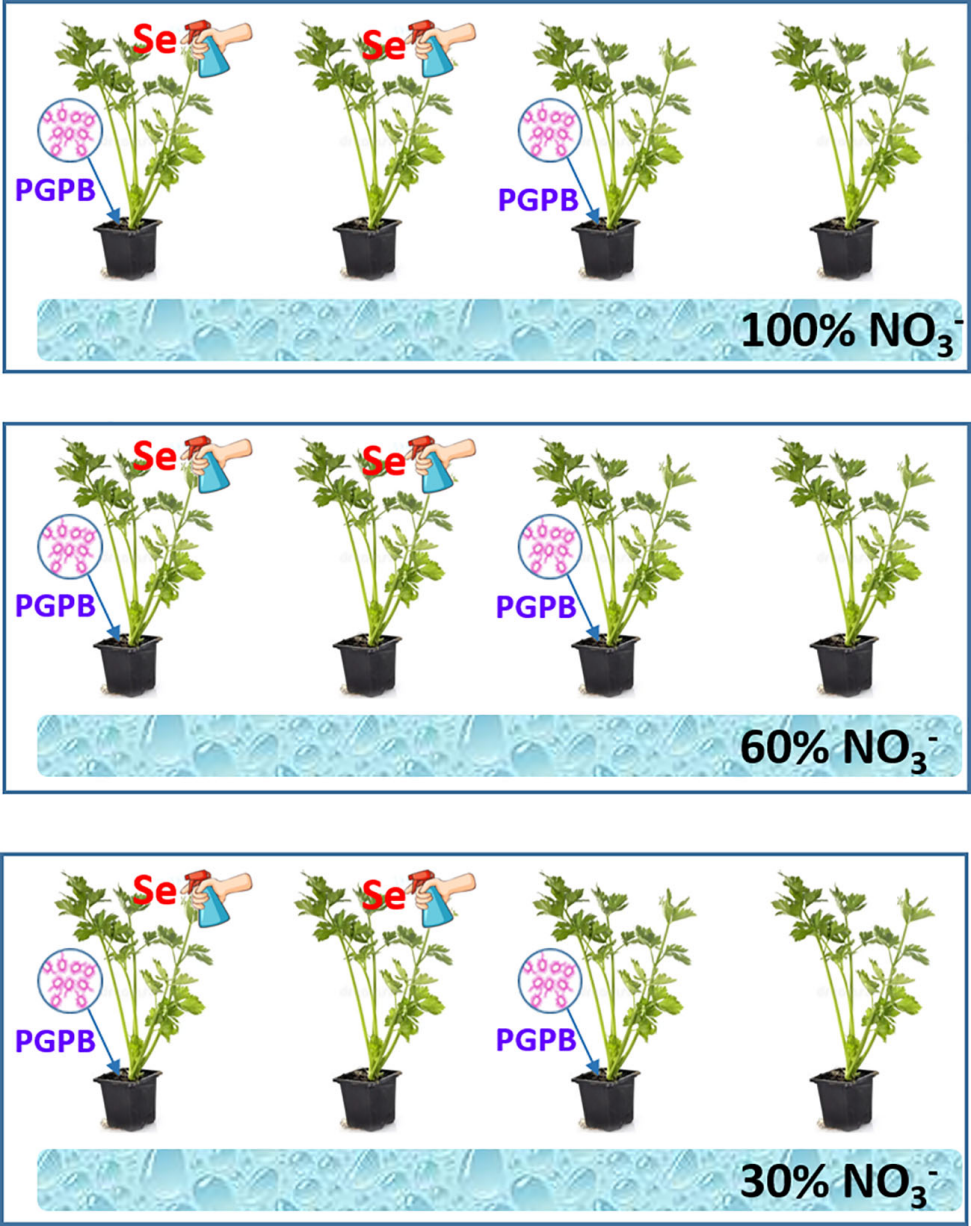
Explanatory diagram of the experimental design.

As can be seen in the **Figure 1**, the irrigation treatments consisted of three fertilizer treatments: twenty four plants were irrigated with treatment 1 (control, 100% of N supply), twenty four plants were fed with treatment 2 (60%, mild deficiency in N supply) and the last group of twenty four plants with the treatment 3 (30%, severe deficiency in N supply). Immediately after transplanting the plants in the plastic bags, half of them (twelve per nutrition treatment) were inoculated with PGPB, specifically with *Azotobacter salinestris* Strain CECT9690 (Ceres Biotics Tech, S. L., Madrid, Spain) (**Figure 1**). Following the manufacturer’s recommendation, the inoculant (10 mL per plant at 250 µg mL^-1^) was applied individually to each pot and mixed homogenously with the substrate. Furthermore, randomly, half of the plants from the inoculated and non-inoculated nutrition treatments were sprayed with a selenium solution. Thus, a total of 12 plants per nutrition treatment (6 inoculated and 6 non-inoculated) were sprayed with Se (**Figure 1**). Se was applied every 15 days. According to work previously carried out by this research group, Se as sodium selenate [Na_2_SeO_4_] was sprayed Se every 15 days at a concentration of 8 μM, as this concentration was shown to provide the best beneficial results for both the health of the plant and its nutritional quality (Piñero et al., 2022).

#### 2.1.3 Sample collection

After 88 days, all the plants were harvested and divided into 2 parts: central and outer (non-edible) parts. The central part was divided into petioles and leaves, while the outer part (both leaves and petioles) was considered as by-product. Consequently, these three parts (petioles, leaves, and by-products) were further divided into three: one part was frozen at -80 °C until the analysis of phenolic compounds and pigments was carried out, the second part was frozen in liquid nitrogen for the enzymatic activity analysis of catalase, and the last was freeze-dried for subsequent analysis of antioxidant activity.

### 2.2 Chemicals and reagents

Authentic standards for gallic acid and 6-hydroxy-2,5,7,8-tetramethylchroman-2-carboxylic acid (Trolox) were acquired from Sigma-Aldrich (Steinheim, Germany). Folin-Ciocâlteu reagent was purchased from Panreac Química (Barcelona, Spain), acetone from Scharlau (Barcelona, Spain) and n-hexane from Macron (Center Valley, PA, USA). The Catalase Colorimetric Activity Kit was obtained from ThermoFisher Scientific (Carlsbad, CA, USA), and ultrapure water was obtained with a Millipore water purification system.

### 2.3 Chlorophylls and β- carotene analysis

Chlorophylls a and b, and β-carotene were analyzed from 1 g of fresh celery leaves, petioles, and by-products after extraction in acetone–hexane (2:3). These pigments were determined spectrophotometrically as described by Nagata and Yamashita (1992) at 663, 645, 505, and 453 nm. Then, the concentrations of chlorophylls a and b, and β-carotene were calculated by using the following equations:


$$\text{Chlorophyll a}\;\left(\text{mg }100{\text{mL}}^{-1}\right)=0.999\;*\;\text{A}663–0.0989\;*\;\text{A}645$$

$$\text{Chlorophyll b}\;\left(\text{mg }100{\text{mL}}^{-1}\right)=-0.328\;*\;\text{A}663+1.77\;*\;\text{A}645$$

$$\text{β}–\text{Carotene}\;\left(\text{mg }100{\text{mL}}^{-1}\right)=0.216\;*\;\text{A}663-1.22\;*\;\text{A}645-0.304\;*\;\text{A}505+0.452\;*\;\text{A}453$$

### 2.4 Antioxidant enzyme activity analysis

The celery samples were ground in mortars with liquid nitrogen to a fine powder (0.2 g), weighted, and homogenized in an extraction buffer (1.5 mL) containing phosphate buffer (50 mmol L^−1^ at pH 7.4), EDTA (1.0 mmol L^−1^), Triton X-100 (0.5%) and polyvinylpolypyrrolidone (PVPP, 10 g) (Aebi, 1984). The homogenates was centrifuged at 11,000 rpm for 20 min at 4°C. After centrifugation, the supernatants were aliquoted into a 96-well plate, and the CAT activity was evaluated using the Catalase Colorimetric Activity Kit (Invitrogen ThermoFisher Scientific, Carlsbad, CA, USA). Following the manufacturer’s protocol, the activity (U/mL) of the antioxidant enzyme was measured at 560 nm using the Synergy MX microplate reader (Biotek Instruments), and quantified with the catalase standard. The enzyme activity was expressed in U g^-1^ FW.

### 2.5 Total phenolic contents analysis

The total phenolic compounds (TPC) were analyzed in celery leaves, petioles, and by-products with the Folin-Ciocâlteu colorimetric method (Kähkönen et al., 1999). Briefly, TPC from fresh samples (0.5 g) were extracted with acetone (80%), and the homogenates were centrifuged at 10,000 × g for 10 min at 4°C. The supernatant (100 µL) was mixed with Folin-Ciocâlteu reagent (1 mL, diluted with Milli-Q water, 1:10) and 2 mL of Milli-Q water, and incubated at room temperature (3 min): Afterward, sodium carbonate (5 mL, 20% *w/v*) was added to the solution and the mixture was re-incubated in the dark at room temperature (1 h). The absorbance of the blue-color resulting from the mixture was measured at 765 nm using a UV–visible spectrophotometer (Shimadzu UV-1800 model with the CPS-240 cell holder, Shimadzu Europa GmbH, Duisburg, Germany). For the quantification, a calibration curve of gallic acid was used, and the results were expressed as gallic acid equivalents (GAE), µg GAE g^−1^ FW.

### 2.6 ABTS antioxidant activity analysis

The antioxidant activity was measured in freeze-dried and milled celery leaves, petioles, and by-products, through the use of the ABTS radical scavenging method. Specifically, lyophilized samples (0.5 g) were homogenized with MeOH/water (80:20, *v/v*) + 1% HCl, sonicated (15 min), and stored at 4 °C (24 h). The mixture was sonicated (15 min) again and centrifuged at 10,000 × g for 10 min at 4°C. The supernatant (10 μL) was mixed with ABTS+* [2,2-azinobis-(3-ethylbenzothiazoline-6-sulphonic acid)] radical cation solution (990 µL), shaken, and incubated in darkness (10 min) (Cano-Lamadrid et al., 2017). Subsequently, the absorbance was measured at 734 nm using a UV–visible spectrophotometer (Shimadzu CPS-240 model, Kyoto, Japan). A Trolox calibration curve (0.01–3 mmol Trolox L^−1^) was used for the quantification of antioxidant activity. The results were expressed as µmol Trolox g^−1^.

### 2.7 Statistical analysis

The experiment was designed based on a completely randomized factorial experiment that corresponded to three N doses supplied (100%, 60%, and 30%), two treatments with PGPB (inoculated and non-inoculated plants), and supply/absence of Se (3 x 2 x 2), and all of the data were analyzed with the SPSS software v.25 (IBM, Chicago, IL, USA). The results are presented as the mean ± standard error of three independent experiments. All data were verified for homogeneity of variance and normality of distribution and were subjected to an analysis of variance (ANOVA) at a 95% confidence level. The mean values were compared with a Tukey’s multiple range test. The analyses of pigments, catalase activity, total phenolic compounds and antioxidant activity were performed using five repetitions (plants) per treatment.

## 3 Results and discussion

### 3.1 Chlorophylls and β-carotene

**Figure 2:**
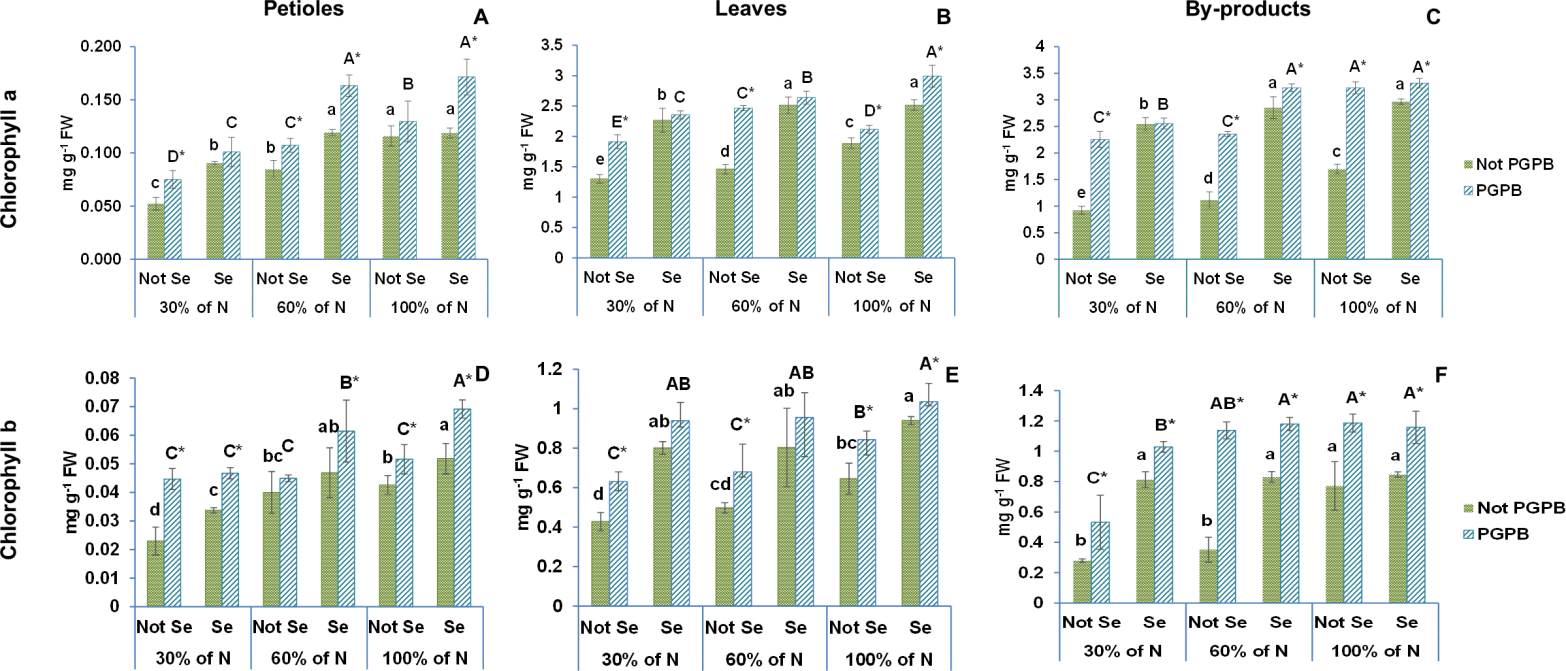
Effect of the combination of inoculation with plant growth promoting bacteria and three different N concentrations (30%, 60% and 100% (control) of N supply) on chlorophylls a and b from celery petioles (**A**, **D**, respectively), leaves (**B**, **E**, respectively) and by-products (**C**, **F**, repectively) from plants sprayed with Se at 8 µM. The data are presented as the treatment means (n = 5) ± standard error. Different small letters indicate significant differences between celery plants in the absence of Se. Different Capital letters indicate significant differences between celery plants sprayed with Se. Asterisks indicate significant differences between inoculated and non-inoculated plants fed with the same N dose and with the same concentration of Se sprayed. Not PGPB, plants non-inoculated with PGPB; Not Se, plants not treated with selenium.

**Figure 3:**
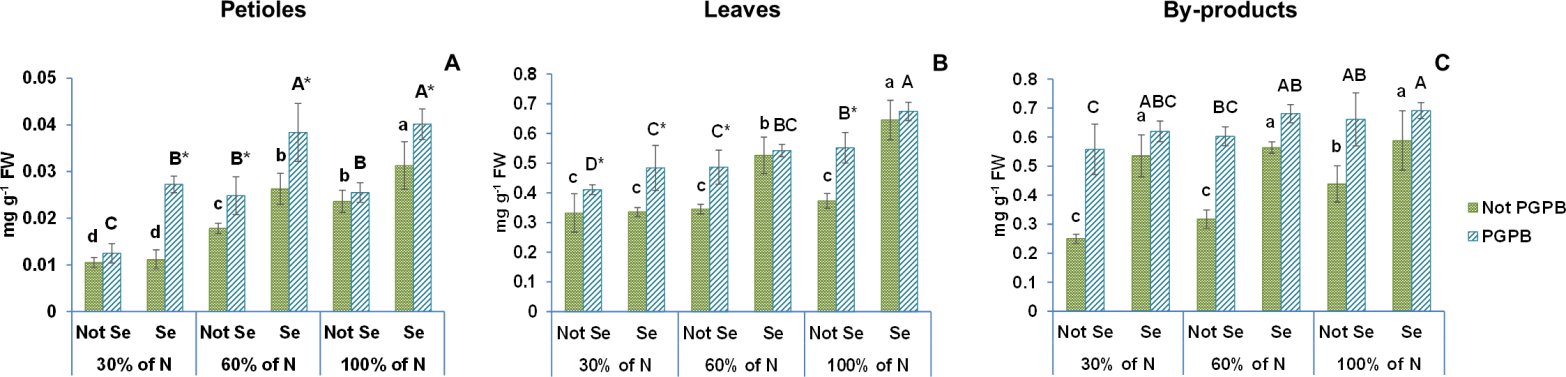
Effect of the combination of inoculation with plant growth promoting bacteria and three different N concentrations (30%, 60% and 100% (control) of N supply) on β-carotene from celery petioles **(A)**, leaves **(B)** and by-products **(C)** from plants sprayed with Se at 8 µM. The data are presented as the treatment means (n = 5) ± standard error. Different small letters indicate significant differences between celery plants in the absence of Se. Different Capital letters indicate significant differences between celery plants sprayed with Se. Asterisks indicate significant differences between inoculated and non-inoculated plants fed with the same N dose and with the same concentration of Se sprayed.

**Figures 2**, **3** show the data obtained for chlorophyll a, chlorophyll b, and β- carotene of celery gladiator variety Innova. The highest chlorophyll b and β- carotene contents were recorded in celery by-products; the highest chlorophyll a content was found in leaves, and the lowest content of all pigments was observed in edible petioles. The chlorophylls a and b contents found were 0.12 mg g^-1^ FW and 0.04 mg g^-1^ FW in petioles, 1.89 mg g^-1^ FW and 0.65 mg g^-1^ FW in leaves, 1.70 mg g^-1^ FW and 1.13 mg g^-1^ FW in by-products, respectively (**Figures 1**, **2**). The β- carotene content oscillated between 0.02 mg g^-1^ FW and 0.44 mg g^-1^ FW depending on the celery part (**Table 1**). These leaves and petioles results are similar with those found in venture, elixir, Samurai, Atlant, and Primus celery varieties (Golubkina et al., 2020; Ingallina et al., 2020; Li et al., 2018).

**Table 1:** ANOVA analysis of chlorophylls, carotene, catalase activity, total phenolic compounds and antioxidant activity under the effect of N dose, inoculation with PGPB and treatment with selenium in isolation or in combination.

Attribute	Part	N dose	PGPB	Se	N x PGPB	N x Se	PGPB x Se	N x PGPB x Se
**Chlorophyll a**	Petioles	***	***	***	*	**	**	**
	Leaves	***	***	***	**	ns	***	***
	By-products	***	***	***	**	***	***	**
**Chlorophyll b**	Petioles	***	***	***	*	ns	*	*
	Leaves	***	***	***	*	ns	*	*
	By-products	***	***	***	***	***	***	**
**β-carotene**	Petioles	***	***	***	ns	ns	***	ns
	Leaves	***	***	***	*	***	**	***
	By-products	***	***	***	ns	*	***	*
**Catalase activity**	Petioles	***	***	***	***	***	ns	*
	Leaves	***	***	***	ns	***	**	**
	By-products	***	***	***	***	***	ns	ns
**TPC**	Petioles	***	***	***	***	***	***	*
	Leaves	***	***	***	***	ns	ns	*
	By-products	***	***	***	***	*	***	**
**Antioxidant activity**	Petioles	***	***	***	ns	*	*	ns
	Leaves	***	***	***	**	***	***	*
	By-products	***	***	***	***	*	ns	ns

Values are the means of five replicate samples; means within columns separated using Tukey’s multiple range test. P = 0.05; n.s. – non significant. *, **, and ***– significant at P ≤ 0.05, 0.005, and 0.001, respectively.

In previous studies carried out on various plant extracts, a relationship between their chlorophyll content and their antimutagenic activity was reported. In fact, *in vitro* and animal studies demonstrated that chlorophylls and their derivatives are potential chemopreventive agents (Ingallina et al., 2020).

The long exposure of celery plants to a nitrogen deficiency led to a significant reduction in the concentration of chlorophylls and β-carotene, with their lowest values observed at the lowest nitrogen dose (**Figures 1**, **2**). These results are in line with the findings by other researchers in lamb`s lettuce and *Castilleja tenuiflora* Benth grown under deficient nitrogen conditions (Collado-González et al., 2023; Medina-Pérez et al., 2015). This gradual degradation in chlorophyll and β-carotene levels may be due to a stimulation of ROS production and oxidative damage caused by N deficiency (Medina-Pérez et al., 2015; Stefaniak et al., 2020). In this situation, plants promote the activity of an antioxidant defense system and the biosynthesis of antioxidant compounds in order to protect their cells against oxidative damage (Stefaniak et al., 2020).

According to the statistics analysis, the nitrogen supply and the PGPB had significant effects on the contents of all pigments in all celery parts. Nevertheless, although the nitrogen supply × PGPB interaction had significant effects on the chlorophyll a and b in all celery parts, the interaction effects on β-carotene content were only observed in leaves (**Table 1**). Inoculation with PGPB significantly increased the concentration of chlorophylls and β -carotene in almost all treatments regardless of the N contribution. In fact, the inoculation with PGPB led to a 43% (30% N supplied) and 27% (60% N) increase in chlorophyll a in petioles, 47% (30% N), 69% (60% N) and 12% (100% N) in leaves, and 145% (30% N), 111% (60% N) and 89% (100% N) in by-products (**Figure 2**). Interestingly, inoculation with PGPB produced a notable increase in the chlorophyll b concentration in all celery parts in plants with a reduced N supply. In this sense, the increase observed in the petiole was 94% (30% N) and 12% (60%); in leaves, 48% (30% N), 37% (60% N); and in by-products, 175% (30% N) and 131% (60% N) (**Figure 2**). The β-Carotene concentration was also significantly higher in PGPB-inoculated plants as compared to non-inoculated plants. Thus, the increase found in petioles was 39% (60% N), in leaves, 41% (60% N) and 48% (100% N), and in by-products, 123% (30% N), 90% (60% N) and 51% (100% N) (**Figure 3**). In previous studies, it has been found that inoculation of plants with *Azospirillum*, *Agrobacterium* and/or *Azotobacter* increases the concentration of chlorophylls in *Rhizophora* seedlings (Jnawali et al., 2015), pea (Ejaz et al., 2020) and maize (Abdel Latef et al., 2020). Ejaz et al. (2020) also reported that inoculation with PGPB could improve the availability of nitrogen in plants, which had a positive effect on the concentration of chlorophylls and carotenoids in plant leaves.

Our data showed that the Se treatment increased the contents of chlorophylls a and b and β-carotene in the different tissues of celery plants (**Figures 2**, **3**). Thus, we observed a 12% increase in the chlorophyll a content in the petioles, 33% in the leaves, and 75% in the by-products. The increase in chlorophyll b content was 22% in petioles, 46% in leaves, and 4% in the by-products. Regarding β-carotene, increases of 33%, 73%, and 55%, were observed in petioles, leaves, and by-products, respectively.

When the combination of Se treatment and N dose was used, significant differences were observed in chlorophyll a in all celery parts, in chlorophyll b in celery by-products, and β-carotene in celery leaves and by-products (**Figures 2**, **3**; **Table 1**). It is known that a Se pretreatment at low concentrations improves the synthesis of chlorophyll and carotenoids in plants under some abiotic stresses, such as salinity and water stress (Ashraf Ganjouii et al., 2023). However, Teixeira et al (Teixeira et al., 2021). reported that chlorophylls and carotenoids were not affected by the combination of selenium application and nitrogen dose in rice. Moreover, Hajiboland et al. (2019), in a work performed with wheat, showed that chlorophyll parameters were significantly reduced at a limited N dose, although those parameters were restored at adequate N levels.

The values of both chlorophylls and β-carotene increased enormously when the selenium treatment was combined with PGPB. In this sense, chlorophyll a values increased by 50% in petioles, by 59% in leaves and 95% in by-products. The chlorophyll b values augmented by 61% in petioles, by 50% in leaves and by 50% in by-products. Likewise, the β-carotene values were improved by 70% in petioles, by 81% in leaves and 58% in by-products.

This is the first time that these three factors are applied in combination in a crop. The ANOVA showed that %N x *Azotobacter salinestris* x Se had significant effects on chlorophyll a and b and β-carotene in the different celery parts (**Table 1**). As a result of the interaction between these the 3 factors, an increase of 93% (both 30% N and 60% N) was observed in chlorophyll a, and 103% (30% N) and 54% (60% N) in chlorophyll b in petioles, as well as an increase of 81% (for 30% N and 60% N) in chlorophyll a, 119% (30% N) and 92% (60% N) in chlorophyll b, and 46% (30% N) and 57% (60% N) in β-carotene in leaves (**Figures 2**, **3**). Regarding the by-products, the increases found were even greater, with increases of 177% (30% N) and 189% (60% N) observed in chlorophyll a, 267% (30% N) and 235% (60% N) in chlorophyll b, and 148% (30% N) and 114% (60% N) in β–carotene (**Figures 2**, **3**). These results are supported by the findings described by Schiavon et al (Schiavon et al., 2020), who reported that the combination of selenium and PGPB greatly increased the amount of chlorophylls and carotenoids in plants under nitrogen deficiency. In fact, these authors claimed that that increase after the Se treatment was much more pronounced than when the Se treatment was applied alone.

### 3.2 Antioxidant enzyme activity: catalase activity

**Figure 4:**
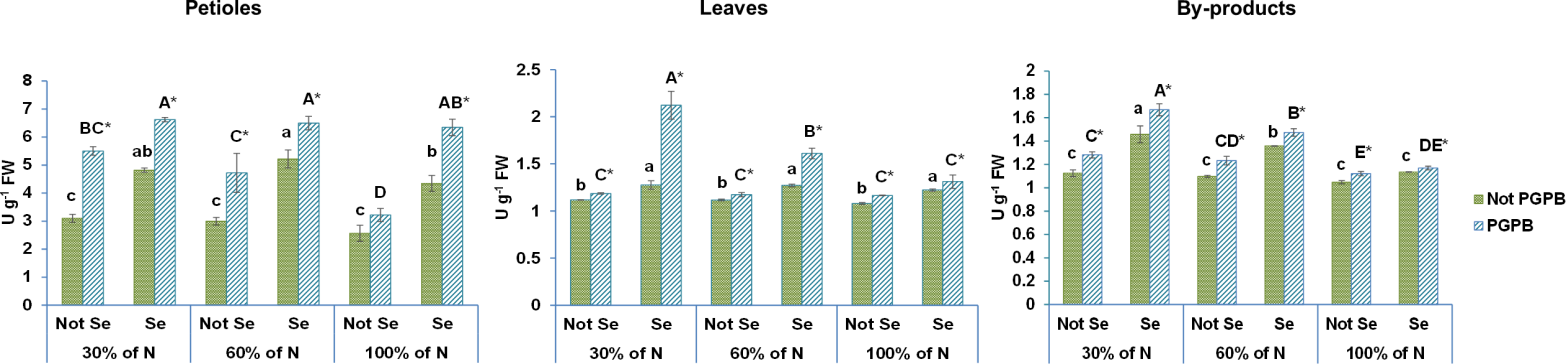
Effect of the combination of inoculation with plant growth promoting bacteria and three different N concentrations (30%, 60% and 100% (control) of N supply) on catalase activity from celery petioles, leaves and by-products from plants sprayed with Se at 8 µM. The data are presented as the treatment means (n = 5) ± standard error. Different small letters indicate significant differences between celery plants in the absence of Se. Different Capital letters indicate significant differences between celery plants sprayed with Se. Asterisks indicate significant differences between inoculated and non-inoculated plants fed with the same N dose and with the same concentration of Se sprayed.

As **Figure 4** shows, no significant differences were observed in CAT activity as the %N provided in the nutrition solution varied. Some authors have reported that depending on plant species and cultivar, the catalase response to the N level can be different. In this sense, an optimum activity of catalase was observed in N-deficient coffee (*Coffea arabica* L.), spinach (*Spinacia oleracea* L. cv. Giant Nobe), rice (*Oryza sativa* L.), tobacco (N. *tabaccum*), mulberry (*Morus alba* L. var. Kanva‐2) and French bean (*Phaseolus vulgaris* L. cv. Strike) plants (Reis et al., 2015). Nevertheless, no effects were observed in catalase activity from N-deficient green leafy kale (*Brassica oleracea* L. var. acephala) (Łata, 2014).

Regarding the effect of *Azotobacter salinestris* on CAT activity, our results showed that plants with this PGPB showed a positive effect in petioles and by-products of celery, as *Azotobacter salinestris* increased the activity of CAT by 25% in petioles and 7% in by-products (**Figure 4**). Similar results were observed in PGPB-treated plants by Agami et al. (2016), who reported that PGPB colonization enhances CAT activity in basil plants (*Ocimum basilicum* L.) under water deficit. Despite the extensive literature on how inoculation with PGPB affects the antioxidants enzymes of plants under saline or water stress (Batool et al., 2020), no information was found on the effects of PGPB inoculation on antioxidant enzymes of plants subjected to N deficiency stress. Although our data showed that a limited N supply alone does not affect CAT activity, when combined with inoculation with PGPB, positive effects were observed. N-deficient plants can exhibit high antioxidant enzyme activities, as they are involved in the detoxification of reduced ROS (Reis et al., 2015). However, the role of antioxidant enzymes is not only to maintain ROS levels low, but they can also regulate ROS-dependent signaling (Noctor et al., 2018). Under optimal growth conditions, ROS are continuously produced, promoting the activities of antioxidant enzymes, such as catalase, that degrade H_2_O_2_ into water and O_2_ (Gebicka and Krych-Madej, 2019). Catalases are considered the main actors in the metabolism of H_2_O_2_ in peroxisomes, in both animals and plants (Noctor et al., 2018). Therefore, the increase in CAT activity is strongly associated with the H_2_O_2_ concentration in N-deficient plants (Noctor et al., 2018). In view of the findings presented in the present study, a greater CAT activity was evident in plants fed with a restricted supply of N (30 and 60%) that were treated with *Azotobacter salinestris*. This finding may perhaps point to a synergistic relationship between N deficiency and the inoculation of plants with this PGPB, which can clearly suggest the positive role of PGPB and limited N support on the activity of antioxidant enzymes, and the subsequent decrease in ROS production in celery plants (Shi et al., 2023). Ahammed et al (Ahammed et al., 2019). reported that antioxidant enzymes control redox homeostasis in most plants. In the literature, it has been described that greater activities of antioxidant enzymes such as APX (ascorbate peroxidase), CAT (catalase), and POD (peroxidase) allows plants to have an increased resistance to abiotic stress (Rajput et al., 2021). According to this, the significant increase in CAT activity as a consequence of the limited dose of N in the plants’ nutrition and inoculation with this type of PGPB, could be indicating that the plant’s antioxidant system has been activated against this nutrition-related abiotic stress, which helps the plant to better overcome the harmful effect of ROS.

Catalase activity increased in all celery parts from plants that received Se at doses of 8 μM, representing an increase of 88% in petioles, 13% in leaves, and 8% in by-products, as compared to the control, respectively (**Figure 4**). Cunha et al. (2023) observed that plants treated with Se, regardless of the dose, showed increased CAT and SOD activity in comparison with the control group. According to Schiavon et al. (2020), the treatment of plants with Se at low doses improves the plant cell antioxidative capacity through the stimulation of antioxidant enzyme activity, such as CAT, POD, and APX, and the biosynthesis of antioxidant compounds, such as phenolic compounds and ascorbic acid. Thus, with the Se treatment, an improvement in the plant’s ability to counteract different types of latent abiotic stress factors was achieved.

Significant %N dose × PGPB x Se results in petioles and leaves from celery plants are shown in **Table 1**. The greatest increase in CAT activity was obtained in all parts of plants fed with the lowest dose of N (30%), inoculated with PGPB, and treated with Se at 8 µM. This result can be explained by the fact that biofortification with Se can be improved with the inoculation of plants with PGPB (Schiavon et al., 2020). In turn, inoculation with an associative bacteria such as PGPB, improves the plant’s tolerance to nitrogen deficiency (Calzavara et al., 2021).

### 3.3 Total phenolic compounds and antioxidant activity

**Figure 5:**
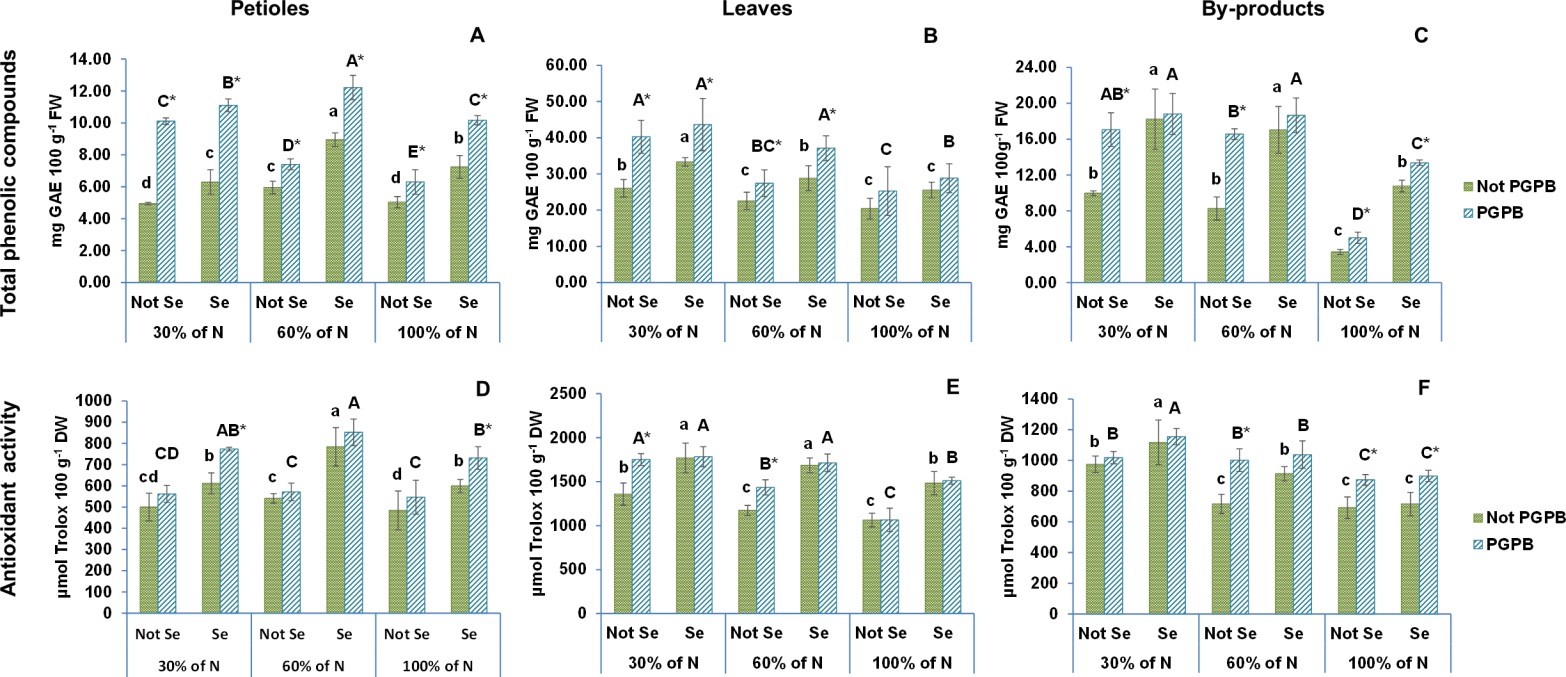
Effect of the combination of inoculation with plant growth promoting bacteria and three different N concentrations (30%, 60% and 100% (control) of N supply) on total phenolic compounds and antioxidant activity from celery petioles (**A**, **D**, respectively), leaves (**B**, **E**, respectively) and by-products (**C**, **F**, respectively) from plants sprayed with Se at 8 µM. The data are presented as the treatment means (n = 5) ± standard error. Different small letters indicate significant differences between celery plants in the absence of Se. Different Capital letters indicate significant differences between celery plants sprayed with Se. Asterisks indicate significant differences between inoculated and non-inoculated plants fed with the same N dose and with the same concentration of Se sprayed.

The results of this study with respect to the total phenolic compounds and antioxidative potential of different parts of raw celery are presented in **Figure 5**. Both the total phenolic compounds and the highest antioxidant activity determined by the ABTS radical scavenging method was found in celery leaves (20.4 mg GAE 100 g^-1^ FW and 1063.1 µmol Trolox 100 g^-1^ DW) followed by by-products (17.0 mg GAE 100 g^-1^ FW and 692.0 µmol Trolox 100 g^-1^ DW), and petioles (5.0 mg GAE 100 g^-1^ FW and 484.8 µmol Trolox 100 g^-1^ DW). As can be observed in our data, the leaves and by-products have a greater antioxidant activity and a higher content of total phenolic compounds than those found in the edible petioles (**Figure 5**). These parameters in the edible petioles portion were higher than those found in the edible petioles of eleven Chinese cultivars by Yao et al (Yao et al., 2010). Taking into account that the petioles and the by-products of celery have a percentage of humidity of 94% and 90, respectively, the total phenolic compounds and the antioxidant activity in our celery by-products was also similar to that obtained by Beltrán Sanahuja et al. (2021) in the by-products of a Spanish cultivar of celery. These authors (Beltrán Sanahuja et al., 2021) proposed a valorization of these by-products as a natural antioxidant food preservative. However, due to the similarity of the results between the edible part and the by-products obtained in this work, the valorization of these by-products should be considered on a larger scale, with the possibility of expanding their application in the cosmetic and nutraceutical sector, to increase their contribution to the circular economy.

The results from the current study show that the highest concentrations of TPC in celery leaves, by-products, and petioles, were observed at the lowest and intermediate N levels, respectively, decreasing as nitrogen supplied increased. This result coincides with the findings obtained by other researchers, who indicated an improved accumulation of polyphenolic compounds in the tissues of various plant matrices as a result of restricted nitrogen nutrition (Munene et al., 2017). According to the vast literature, there is a negative relationship between the accumulation of polyphenolic compounds and the restriction of N in plant nutrition (Stefaniak et al., 2020). Thus, while a greater N availability favors the primary growth of plants at the expense of secondary metabolism, a limited amount of N can lead to a greater availability of phenylalanine, enhancing the biosynthesis of flavonoids. The primary and secondary metabolic pathways of plants are linked by phenylalanine ammonia-lyase (PAL) (Stefanelli et al., 2010). In situations of N deficiency, PAL releases N for redistribution in plants, making it a key enzyme in circumstances of nutritional stress due to N deficiency (Medina-Pérez et al., 2015). Although most of the crops studied produced the highest content of phenolic compounds under the greatest nitrogen restriction, some exceptions exist, such as bilberries, onions, and olives, which did not show any effect on phenolic compounds in N-starved plants. In fact, there are currently no studies that indicate an optimal N application rate in plant nutrition that allows achieving a balance between yield and the optimal content of polyphenolic compounds, such as flavonoids (Stefanelli et al., 2010).

Due to the fact that phenolic compounds belong to the many groups of molecules that contribute the most to antioxidant activity, it is logical to assume that, as shown in **Figure 5**, the antioxidant capacity shows the same trend as the total phenolic compounds. Similar findings have been observed in lamb’s lettuce (Collado-González et al., 2023), kiwiberry (Stefaniak et al., 2020), and amaranth (Munene et al., 2017). Based on these findings, Stefanelly et al. (2010) indicated that a good strategy to stimulate the production of compounds with a high antioxidant activity could be the manipulation of N availability.

The inoculation with the *Azotobacter salinestris* bacteria produced plants with higher TPC and antioxidant activity values (**Figure 5**). Indeed, the highest TPC and antioxidant activity in celery leaves and by-products were observed in those plants fed with the lowest nitrogen dose and inoculated with *Azotobacter salinestris*, and the highest TPC and antioxidant activity of celery petioles were found in those plants that were also inoculated with that bacteria and fed with a contribution of 60% of N to their nutritional solution. In any case, the lowest TPC and antioxidant activity were obtained in plants not inoculated with this PGPB and fed with the highest N dose (**Figure 5**). An increase in phenolic acids and antioxidant activity in response to biostimulants, as the PGPB, has also been reported in extracts of different tissues (Chiappero et al., 2021).

The antioxidant activity of the by-products showed a remarkable increase in all celery parts due to the application of Se. Similar results were observed by several authors, who reported a significant increase in the total polyphenolic compounds and antioxidant activity in several plant species by using Se fertilizer *(* Marfetán et al., 2023), with Se also playing an important role in increasing the antioxidant capacity (Ashraf Ganjouii et al., 2023).

Furthermore, a synergistic effect between the 3 factors studied (the reduction in the N dose, the inoculation with PGPB, and the application of selenium) was observed on TPC in all celery parts. In contrast, and as observed with the CAT activity results, the interaction of these 3 factors only had significant effects on celery leaves and petioles.

## 4 Conclusions

The results from the current work showed an accumulation of TPC (total phenolic compounds) and antioxidant activity as N dose was reduced. However, pigments (chlorophylls and β-carotene) showed a different behavior when the N dose decreased, and CAT activity was not affected by the N dose. Our data revealed a build-up of TPC, and increases in antioxidant activity, antioxidative enzymatic activity, and pigments, when a reduced N dose was supplied to the celery plants inoculated with Azotobacter salinestris. Furthermore, Se at doses of 8 μM reinforced the beneficial effect of the synergistic interaction between N dose and PGPB in celery plants.

Thus, this novel and sustainable strategy composed of N dose, inoculation with PGPB (*Azotobacter salinestris*), and the treatment with Se, should be considered as a good strategy for stimulating the production of antioxidant activity and antioxidants compounds in plants.

On the other hand, the leaves and by-products showed the highest content of bioactive compounds with a greater antioxidant capacity. Based on these results, we paved a way to develop new processes and methods to obtain high added value products from waste residues of the cropping system.

## Data Availability

The original contributions presented in the study are included in the article/supplementary material. Further inquiries can be directed to the corresponding authors.
